# Nanomechanical properties of distinct fibrillar polymorphs of the protein α-synuclein

**DOI:** 10.1038/srep37970

**Published:** 2016-11-30

**Authors:** Ali Makky, Luc Bousset, Jérôme Polesel-Maris, Ronald Melki

**Affiliations:** 1 Institut Galien Paris-Sud, CNRS, Univ. Paris-Sud, University Paris-Saclay, 92296 Châtenay-Malabry, France; 2 Paris-Saclay Institute of Neuroscience, Centre National de la Recherche Scientifique, Université Paris-Saclay, 91190 Gif-sur-Yvette, France; 3 Luxembourg Institute of Science and Technology (LIST), Materials Research and Technology (MRT), L-4422 Belvaux, Luxembourg

## Abstract

Alpha-synuclein (α-Syn) is a small presynaptic protein of 140 amino acids. Its pathologic intracellular aggregation within the central nervous system yields protein fibrillar inclusions named Lewy bodies that are the hallmarks of Parkinson’s disease (PD). In solution, pure α-Syn adopts an intrinsically disordered structure and assembles into fibrils that exhibit considerable morphological heterogeneity depending on their assembly conditions. We recently established tightly controlled experimental conditions allowing the assembly of α-Syn into highly homogeneous and pure polymorphs. The latter exhibited differences in their shape, their structure but also in their functional properties. We have conducted an AFM study at high resolution and performed a statistical analysis of fibrillar α-Syn shape and thermal fluctuations to calculate the persistence length to further assess the nanomechanical properties of α-Syn polymorphs. Herein, we demonstrated quantitatively that distinct polymorphs made of the same protein (wild-type α-Syn) show significant differences in their morphology (height, width and periodicity) and physical properties (persistence length, bending rigidity and axial Young’s modulus).

Parkinson’s disease (PD) is the second most frequent neurodegenerative disease in human after Alzheimer’s disease^1^. It is characterized by the abnormal intracellular aggregation of the 140 amino acid residues presynaptic protein alpha-synuclein (α-Syn) into fibrillar assemblies that are the main constituents of Lewy bodies^2^,^3^,^4^. The seminal observation made by Heiko Braak and co-workers on autopsy cases that Lewy pathology initiates in circumscribed areas of the brain and progresses in a topographically predictable manner following anatomical connections^5^ together with the finding that Lewy bodies in PD brains contaminate grafted fetal mesencephalic progenitor neurons decades after transplantation^6^,^7^ suggest that Lewy bodies have prion-like properties. Since then, a plethora of *in vivo* and *in vitro* studies have established the ability of Lewy bodies and preformed high molecular weight assemblies of α-Syn, in particular those of fibrillar nature, to induce Lewy bodies-like pathology and propagate from one cell to another and/or within the central nervous system in a prion-like manner^1^,^8^,^9^,^10^,^11^,^12^,^13^,^14^,^15^,^16^,^17^,^18^,^19^,^20^.

In solution, pure α-Syn adopts an intrinsically disordered structure^21^ and assembles into fibrils that exhibit considerable morphological heterogeneity^1^,^22^,^23^. Such polymorphism may lead to significant differences in fibrils height, width, periodicity, length and mechanical properties. Indeed, morphologically distinct α-Syn fibrils with “curly shape” or “straight shape” have been shown to exhibit persistence lengths of 0.17 and 140 μm, respectively^24^. We recently established tightly controlled experimental conditions allowing the assembly of α-Syn into highly homogeneous and pure polymorphs. We also showed that distinct polymorphs not only differ in shape as assessed by transmission electron microscopy but also in their intrinsic structure as assessed by solid-state NMR and limited proteolysis and their functional properties (e.g. binding to cells, toxicity and seeding capacities) as assessed by a variety of cell biological assays^1^. We further demonstrated that upon intra-cerebral injection to rodents, distinct polymorphs could be distinguished by their ability to yield either Lewy bodies- and Lewy neurites-like deposits, the hallmarks of Parkinson’s disease or Lewy bodies- and Lewy neurites-like deposits and glial cytoplasmic inclusions in oligodendroglia, the hallmark of multiple system atrophy^1^,^20^. Thus, our findings establish structural-molecular basis for distinct synucleinopathies^1^,^20^.

To further document the physical properties of the two polymorphs we characterized structurally and functionally and that of two additional polymorphs we generated, we have imaged the four fibrillar α-Syn polymorphs by atomic force microscopy (AFM) in air and performed a statistical analysis of the shape and thermal shape fluctuations^25^,^26^,^27^,^28^ on two different substrates to take into account the possible effect of the underlying substrate to fibrils properties^29^. We demonstrate here that distinct fibrillar strains made of the same protein differ very significantly by their physical properties.

## Results

### Morphological analysis of α-Syn fibrillar assemblies

**Figure 1:**
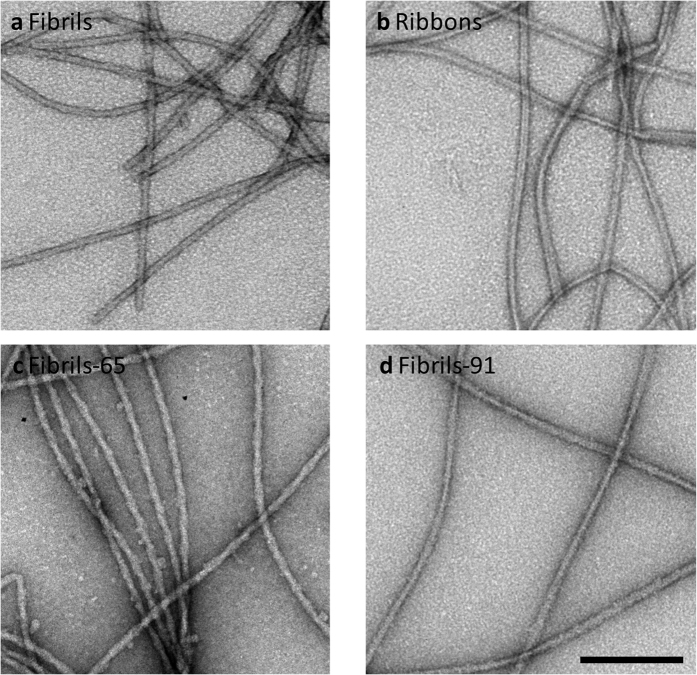
TEM images of the different α-Syn fibrillar polymorphs. Electron micrographs of α-Syn (**a**) fibrils, (**b**) ribbons, (**c**) fibrils-65, (**d**) fibrils-91 assemblies adsorbed onto carbon coated copper grid stained with Uranyl acetate. Scale bar 200 nm.

*In vitro*, under neutral pH and physiological ionic strength, α-Syn assembles into a multitude of mega-dalton particles with fibrillar shapes. Some fibrils appear twisted (left or right handed twists), while some are not^30^. When twists are apparent, the pitch varies significantly from one fibril to another and even within one given particle. Furthermore, some fibrils appear thick, while others appear thin^31^. The abovementioned heterogeneity is incompatible with a straightforward and statistically meaningful characterization of the physical properties of populations of fibrillar α-Syn. As the assembly conditions of α-Syn have been repeatedly shown to influence the nature of the fibrillar particles that form and their homogeneity^1^,^22^,^23^,^30^,^32^, we revisited α-Syn assembly into fibrils under tightly controlled experimental conditions. We recently generated two structurally distinct, highly homogeneous, fibrillar α-Syn assemblies^1^. The homogeneity of the structurally distinct fibrillar assemblies allowed demonstrating that they possess different functional properties *in vitro*^1^ and *in vivo*^20^. Transmission electron microscopy analysis reveals that while the polymorph generated at pH 7.5 and in the presence of 150 mM KCl has a cylindrical aspect (Fig. 1a), that obtained at the same pH but under low salt conditions (<0.1 mM NaCl) or in the presence of 150 mM KCl and 2.5 mM EDTA is flat and exhibits twists (Fig. 1b). We will refer throughout this work to the cylindrical assemblies as fibrils and to the flat assemblies as ribbons. Under slightly acidic conditions and physiological ionic strength concentration (pH 6.5 and 150 mM NaCl), mimicking lysosomal environment, α-Syn forms fibrillar structures that are cylindrical, thus resembling fibrils, but that look slightly thicker in the electron microscope (Fig. 1c). Under basic and low ionic strength conditions (20 mM KPO_4_, pH 9.1), α-Syn forms fibrillar structures that are cylindrical with a fuzzy surface (Fig. 1d). We will refer throughout this work to the assemblies that form at pH 6.5 and pH 9.1 as fibrils-65 and fibrils-91, respectively. Cross-section analysis of the different fibrillary polymorphs performed on negatively stained TEM shows that fibrils and fibrils-65 exhibit similar and constant widths, 14.1 ± 1.3 nm s.e.m., n = 80 and 14.4 ± 1.3 nm, respectively, while ribbons and fibrils-91show wide and narrow widths, 12.5 ± 0.9 nm and 17.8 ± 1.6 nm s.e.m., n = 80 for ribbons, 15.2 ± 2.7 nm and 19.0 ± 1.5 nm s.e.m., n = 80 for fibrils-91 (Fig. S1).

To determine whether the morphological differences observed in the electron microscope translate into differences within the physical properties of the elongated particles, we imaged the different hydrated fibrillar assemblies in ambient air following their adsorption onto mica or HOPG surfaces by AFM at the highest possible resolution. We next derived the persistence length and bending rigidity of the different fibrillar assemblies.

**Figure 2:**
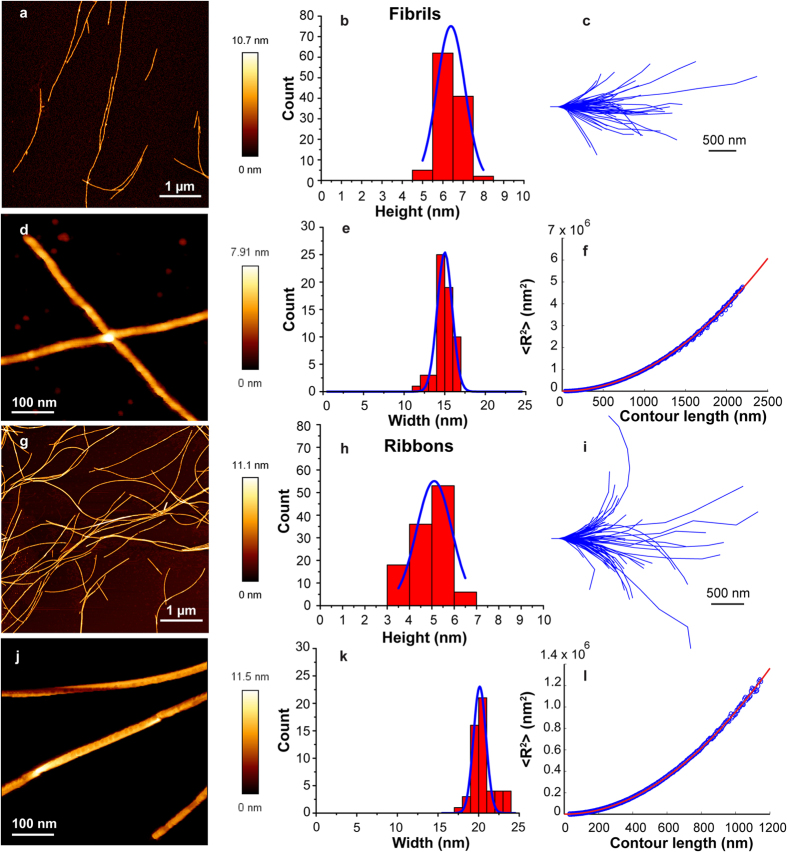
(**a,g**) AM-AFM topography images in air mode of hydrated α-Syn fibrils and ribbons, respectively, adsorbed onto mica substrate. These images were used for fibrils height determination. (**b,h**) Histograms of fibrils and ribbons height distributions. (**c,i**) Contours of fibrils and ribbons imaged by AFM, where initial tangents were aligned. (**d,j**) PM-AFM topography images of α-Syn fibrils and ribbons, respectively, obtained with Ultrasharp probes in air mode. These images were used for fibrils width determination. (**e,k**) Histograms of α-Syn fibrils and ribbons width distributions. (**f,l**) End-to-end distance (R^2^) plots as a function of contour length for α-Syn fibrils and ribbons, respectively, adsorbed on mica (blue open circles). Least-square fits are shown as red lines.

**Figure 3:**
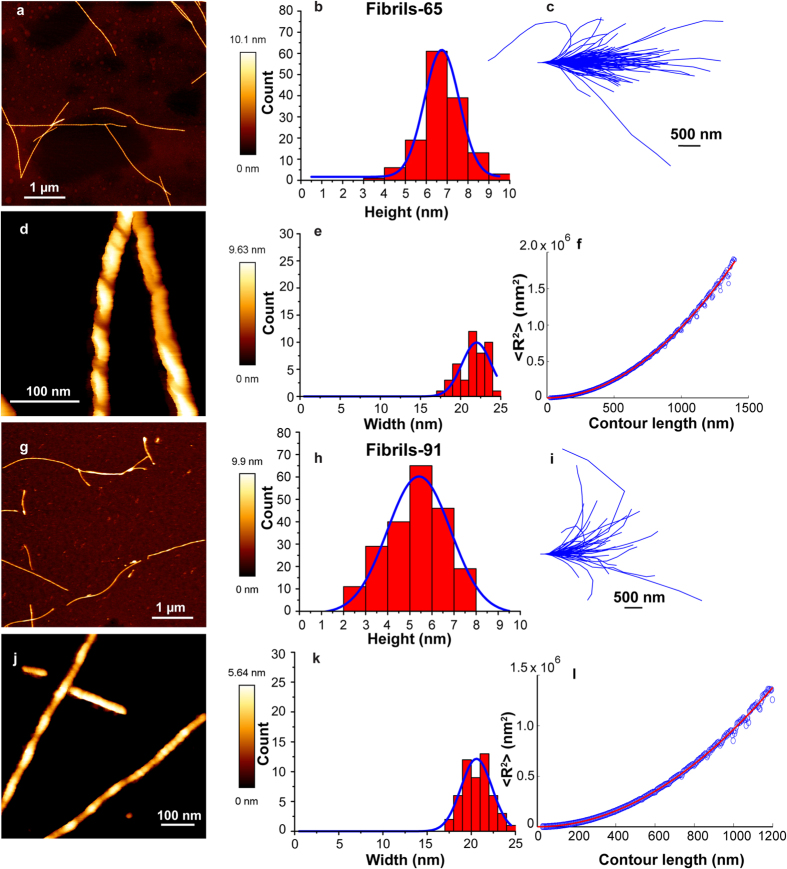
(**a,g**) AM-AFM topography images in air mode of hydrated α-Syn fibrils-65 and -91, respectively, adsorbed onto mica substrate. These images were used for fibrils-65 and -91 height determination. (**b**,**h**) Histograms of fibrils-65 and -91 height distributions. (**c,i**) Contours of fibrils-65 and -91 imaged by AFM, where initial tangents were aligned. (**d,j**) PM-AFM topography images of α-Syn fibrils-65 and -91, respectively, obtained with ultrasharp probes in air mode. These images were used for fibrils-65 and -91 width determination. (**e,k**) Histograms of α-Syn fibrils-65 and -91 width distributions. (**f,l**) End-to-end distance (R^2^) plots as a function of contour length for α-Syn fibrils-65 and -91, respectively, adsorbed on mica (blue open circles). Least-square fits are shown as red lines

AM- and PM-AFM topography images of several μm long fibrils, ribbons, fibrils-65 and fibrils-91 are displayed in Figs 2a,d,g,j and 3a,d,g,j respectively. The height, width and periodicity, when present, for each fibrillar type was determined first. Whereas α-Syn fibrils have a mean height of 6.4 ± 0.7 nm (n = 110) and a width of 15 ± 0.9 nm (n = 61) (Fig. 2b,e), the measured height and width of ribbons were 5.1 ± 0.8 nm (n = 113) and 20.1 ± 0.7 (n = 53) nm (Fig. 2h,k), respectively. The values we obtained using 3 distinct monomeric α-Syn preparations are statistically significant as determined by the unpaired two sample Student’s t-test, *P* < 0.001 and are in good agreement with those we derived from cryo-electron microscopy observations 13 ± 2 nm and 18 ± 1.5 nm for the wide sections of fibrils and ribbons, respectively^1^.

While AFM imaging of fibrils and ribbons with conventional cantilevers did not reveal any detectable periodic height variation along their length or any significant morphological difference at large scale (Fig. 2a,g), the high resolution AFM images performed with ultrasharp probes in PM-AFM (Fig. 2d,j) allowed the detection of different features between the two strains. α-Syn fibrils did not show clear periodic height fluctuations along their axis, but they exhibited instead small irregular ones (Figs S2 and 2d) which were slightly higher than the AFM noise level. Interestingly, these irregular fluctuations were sometimes associated with some small periodic ones as shown in Fig. 2d. α-Syn ribbons exhibited some periodic holes along their axis (Fig. 2j). In addition, ribbons exhibited twists (Fig. 2j) in agreement with previous observations we made using transmission and cryo-electron microscopy^1^.

The AM- and PM-AFM topography images of fibrils-65 and fibrils-91 are displayed in Fig. 3. The mean heights of fibrils-65 and fibrils-91 are 6.7 ± 0.8 nm (n = 144) and 5.4 ± 1.5 nm (n = 210), respectively. This difference in height (~18%) is similar to that between fibrils and ribbons and is statistically significant P < 0.001 (unpaired two sample Student’s t-test). Fibrils-65 and fibrils-91 have similar average width 21.9 ± 1.8 nm (n = 45) and 20.6 ± 1.7 nm (n = 52), respectively. Altogether our observations suggest that whereas ribbons, fibrils-65 and fibrils-91 polymorphs exhibit similar widths (~5% variation), the width of the fibrils polymorph is ~28% lower. Furthermore, fibrils-65 polymorph exhibited the largest height and width.

**Figure 4:**
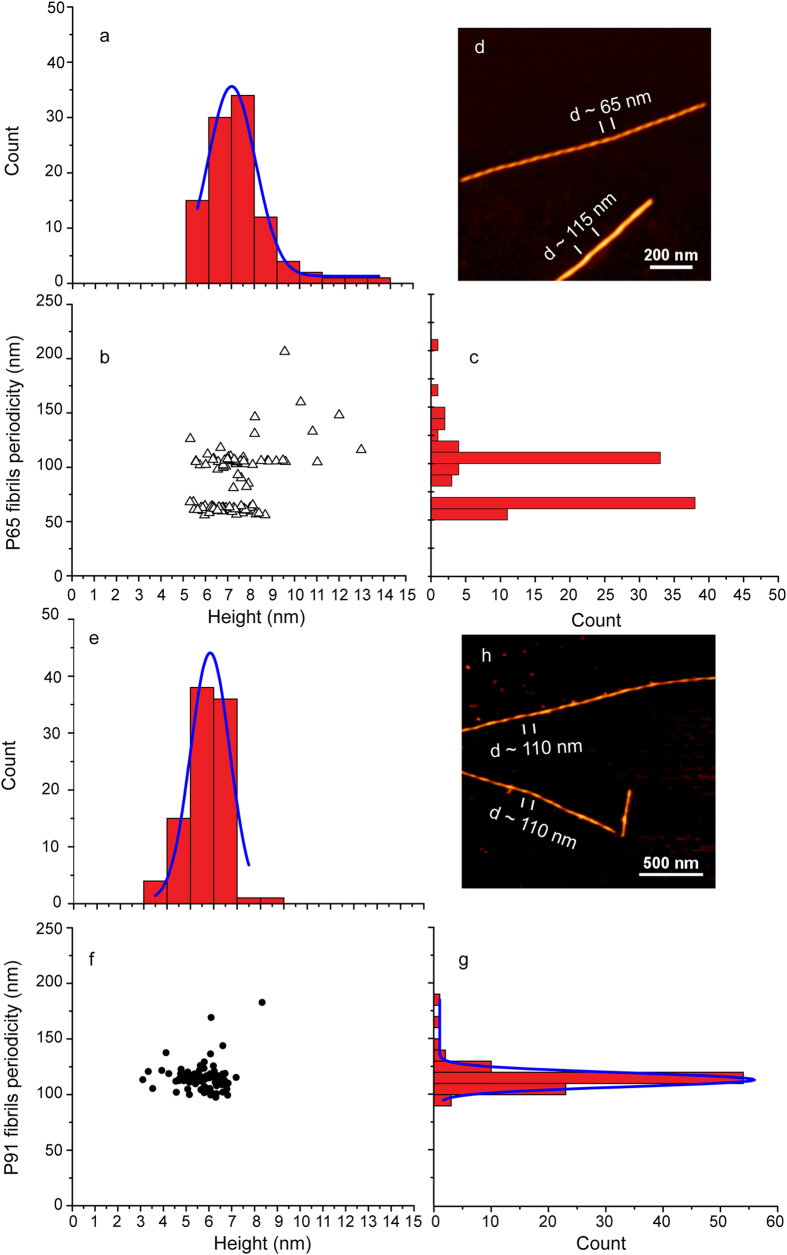
Scatter plots of α-Syn fibrils-65 and -91 periodicities are depicted in (**b**,**f**), respectively. Next to each plot are presented the histograms with the Gaussian fit of the periodicities distribution. The histograms of the height (**a,e**) and periodicity (**c,g**) distributions with the Gaussian fit (blue line) are also depicted. AFM images showing representative periodicities observed for α-Syn fibrils-65 (**d**) and fibrils-91 (**h**).

High resolution AFM imaging of fibrils-65 and fibrils-91 with ultrasharp probes in PM-AFM (Fig. 3d,j) revealed clear periodic height fluctuations along their length. The periodicity was thus analyzed quantitatively for both types of fibrils along at least 600 nm of length and the results are shown in Fig. 4. Interestingly, fibrils-65 and -91 exhibited different height periodicities. Moreover, the periodicity of fibrils-65 exhibited a broad distribution and appeared to have two distinct components with average values of ~60 nm and ~105 nm. Fibrils-91 showed a narrower distribution of periodicity with an average value of ~113 nm. The difference in height periodicities between the distinct fibrillar assemblies was preserved when HOPG substrate was used for imaging (Fig. S3), we therefore conclude that these differences are not related to the substrate.

Average height periodicities of 50, 100 and 150 nm have been reported for fibrillar assemblies made of wild type and A140C mutant α-syn in 10 mM of Tris buffer at pH 7.4 and in the absence of EDTA^22^. The same authors reported one dominant population with average height periodicity of 100 nm in the presence of EDTA^22^. Our observations suggest that beside divalent ions, pH and the ionic strength affect the overall shape and periodicity of α-syn fibrillar assemblies.

A relationship between fibrils periodicity and height for insulin^33^ and β-lactoglobulin^34^ fibrillar assemblies has been reported and a hierarchical assembly model (HAM) has been proposed to account for this observation^35^. To determine whether a relationship between fibrils periodicity and height exists in α-Syn fibrillar assemblies, we have plotted the variation of periodicity as a function of fibrils height (Fig. 4). The plot for fibrils-65 (Fig. 4b) shows two different clusters of periodicity while only one cluster is observed for fibrils-91 (Fig. 4f). Fibrils-65 displayed two distinct periodicities and a narrow height distribution while fibrils-91 exhibited a narrow height distribution with a single periodicity cluster. Thus, no correlation between the height and periodicity could be observed for α-Syn fibrils exhibiting clear periodicity e.g. fibrils-65 and -91. Overall, our results agree with those obtained by Sidhu *et al*.^22^ on wild-type α-Syn fibrillar assemblies showing that the morphological parameters of height and periodicity in mature α-Syn fibrils are not coupled.

### Nanomechanical properties of the different α-Syn fibrillar polymorphs

We next assessed the nanomechanical properties of the different α-Syn fibrillar assemblies we generated. To this aim, we performed a statistical analysis of the shape thermal fluctuations of the different α-Syn fibrillar assemblies adsorbed onto mica substrate and imaged with AFM in air conditions. The contour length of isolated fibrillar assemblies from topographical images were tracked automatically using Easyworm software^25^ (see Figs 2c,i and 3c,i) and the persistence length (P_*l*_) was calculated using the end-to-end distance approach derived from the WLC model for semi-flexible polymers. Since the P_*l*_relates to the length above which the thermal energy can bend a semi-flexible polymer, its determination was essential for the bending rigidity calculation. Figures 2(f,l) and 3(f,l) show the mean square end-to-end distance <R^2^> plots as the function of the contour length for fibrils, ribbons, fibrils-65 and -91, respectively. The calculated P_*l*_, listed in Table 1, demonstrated that the distinct fibrillar assemblies exhibit different mechanical properties. α-syn fibrils showed the highest P_*l*_(14.2 ± 3.3 μm), followed by fibrils-65 and -91 with a P_*l*_ of 6.5 ± 3.2 μm and 5.8 ± 1.7 μm, respectively. α-Syn ribbons exhibited the lowest P_*l*_ value of 3.5 ± 1.0 μm. In addition, the bending rigidity followed the same tendency suggesting that α-Syn fibrils were the most rigid fibrillar assembly with a bending rigidity of ~5.8 × 10^−26^ N.m^2^, a value ~4 folds higher than that of ribbons and ~2 folds higher than those of fibrils-65 and -91. Such values of bending rigidities are in accordance with those obtained for amyloid proteins determined with the same technique (1.4 × 10^−28^ N.m^2^−1.3 × 10^−24^ N.m^2^)^36^.

The persistence length we measured in this work are significantly lower than those reported by other authors using the same technique but different experimental conditions. Indeed, Bhak *et al*.^24^ have reported persistence lengths of up to 140 μm for one fibrillar polymorph of wild-type α-Syn, whereas Sweers *et al*. have obtained values of ~360 μm for a fibrillar form of α-syn E46K variant associated to familial early onset Parkinson’s disease^37^. Such differences in values may be due to the intrinsic nature of assemblies formed under distinct experimental conditions but also accounted for either by the underlying substrate, the imaging conditions (in air or liquid) or the fibrillar assemblies that did not reach 2D equilibrium when the imaging and statistical analysis were performed. In the present study the fibrils were adsorbed overnight onto freshly cleaved mica to make sure they reached 2D equilibrium. As imaging in liquid conditions was not possible due to the weak adsorption of fibrils on mica in buffer conditions, we imaged the fibrils in air. As fibrils persistence length depends not only on their intrinsic mechanical properties but also on the underlying substrate and the preparation conditions of the samples, in particular water evaporation process, we used two different substrates and very stringent adsorption/water evaporation conditions. The tightly controlled experimental conditions we used ensured hydration after overnight adsorption to the substrate of the different fibrillary assemblies. We also compared the P_*l*_ we measured upon adsorption of the distinct fibrillar assemblies we generated onto mica or HOPG. Mica is a hydrophilic substrate with a high surface energy in ambient conditions (130–170 mJ/m^2^)^38^ while HOPG is hydrophobic with lower surface energy (~70 mJ/m^2^)^39^. The P_*l*_ values derived from distinct α-syn fibrillar assemblies imaged onto HOPG (Fig. S4) and mica were similar (Table 1). These results indicated thus an independence of the persistence length from the substrate type, also that the fibrils were fully equilibrated in 2D conformation under our experimental conditions.

For the axial Young’s modulus determination, we calculated the second moment of area (*I*) from the average heights and widths of fibrils (Table 1). Based on their cross sectional measurements (width > height) two models could be attributed to the imaged fibrils either “tape-like” or “cylinder-like” assemblies with rectangular or ellipsoidal cross sections, respectively. In this work, we assumed that the fibrillar α-Syn cross section has an ellipsoidal shape (eq. 4) although the polymorph named ribbons exhibited a “tape-like” shape as demonstrated from previous transmission and cryo-electron microscopy observations^1^. This is because the “tape-like” model assumes a strictly rectangular cross-section and does not take into account the circular or ellipsoidal shape of the protofibrils^35^ leading to an overestimation of the second moment of area and thus an underestimation of their Young’s modulus. The calculated axial young modulus of the different polymorphs (Table 1) shows that α-syn fibrillar assemblies exhibit low moduli of elasticity ranging from 0.08 to 0.3 GPa. The young modulus values were even lower when the “tape-like” model was used for the calculation of the second moment of area (Table 1). The values we determined were two orders of magnitudes lower than those reported in the literature for other amyloid fibrils such as insulin^36^,^40^, Aβ peptide^36^, β-lactoglobulin^28^,^36^,^41^, transthyretin^36^ and E46K α-Syn fibrils (16–24.7 GPa)^37^ using the same method. However the calculated axial young moduli are in the same order of magnitude of mouse prion protein amyloid fibrils that are known for their high intrinsic mechanical flexibility^29^. This result suggests that α-Syn fibrillar assemblies are less resistant to bending than other amyloid fibrils. This may reflect characteristic intermolecular interactions misfolded α-Syn monomers establish within fibrils.

## Discussion

The aim of this work was to assess the morphological and nanomechanical properties of homogenous and pure α-Syn polymorphs that were assembled in tightly controlled conditions. To do so, the morphology of four homogeneous α-Syn polymorphs were characterized with AFM at high resolution and their height, width and periodicity determined. In addition, the nanomechanical properties of the distinct α-Syn fibrillary polymorphs were quantitatively determined with a statistical analysis of their shape thermal fluctuations using two different substrates with distinct surface energy in order to take into account the possible effect of the underlying substrate. Interestingly, the four polymorphs did not show the same morphology and exhibited different mechanical properties. Indeed, while fibrils-65 and fibrils-91 were periodic, fibrils and ribbons did not show any periodicity with twists observed in the case of ribbons. Moreover, the four fibrillar polymorphs differed significantly in their height and width. The persistence length calculation for the four α-Syn polymorphs revealed significant differences in their axial Young’s modulus regardless of the underlying substrates. Although we did not directly measure α-Syn polymorphs radial modulus, the significant differences between the axial moduli of the different fibrillary assemblies we generated suggest they exhibit distinct nanomechanical characteristics. Indeed, while a difference between the axial and radial elastic moduli of α-Syn fibrillar polymorphs may exist, such difference would be slight as demonstrated previously for amyloid fibrils^28^,^29^. Thus, our results bring valuable insight into the morphological and nanomechanical characteristics of four different fibrillar α-Syn strains.

## Methods

### Expression and purification of α-Syn

Recombinant wild-type α-Syn was expressed in Escherichia coli strain BL21(DE3) (Stratagene, La Jolla, CA, USA) transformed with the expression vector pET3a (Novagen) encoding wild-type, full-length α-Syn. The expression of α-Syn was induced by 0.5 mM IPTG for 2 h when the bacteria grown in LB medium at 37 °C reached an optical density of 1.0 at 660 nm. Soluble, monomeric α-Syn was purified from the bacteria lysate as previously described^42^. α-Syn concentration was determined spectrophotometrically using an extinction coefficient of 5960 M^−1^.cm^−1^ at 280 nm. Pure α-Syn (0.2–0.5 mM) in 50 mM Tris-HCl, pH 7.5, 150 mM KCl was filtered through sterile 0.22-μm filters and stored at −80 °C.

### Preparation of different assemblies of a-syn

α-Syn in different buffers, (see below) was incubated one week at 37 °C under continuous shaking in an Eppendorf Thermomixer set at 600 r.p.m. to assemble into distinct fibrillar polymorphs. To obtain the polymorph “fibrils”, α-Syn (400 μM) was assembled in 50 mM Tris-HCl, pH 7.5, 150 mM KCl buffer^1^; to obtain the polymorph “ribbon”, α-Syn (400 μM) was dialyzed overnight against 5 mM Tris-HCl pH 7.5 prior to assembly^1^; to obtain the polymorph “fibrils-91”, α-Syn (400 μM) was dialyzed overnight against 20 mM KPO_4_ pH 9.1 prior to assembly^43^; to obtain the polymorph “fibrils-65”, α-Syn (400 μM) was dialyzed for 3 hours against 20 mM MES pH 6.5, 150 mM NaCl prior to assembly.

### Transmission electron microscopy

The nature of fibrillar α-Syn strains was assessed using a JEOL 1400 transmission electron microscope following adsorption onto carbon-coated 200-mesh grids and negative staining with 1% uranyl acetate. The images were recorded with a Gatan Orius CCD camera (Gatan).

### Atomic force microscopy imaging

For AFM imaging, the different fibrillar α-Syn strains were first diluted in milli-Q water to a final concentration of ~5 μg/ml. Afterwards, 100 μl of the diluted fibrils solutions were deposited on either freshly cleaved mica (muscovite mica, grade V1 from Tedpella) or HOPG (Highly Ordered Pyrolytic Graphite, grade ZyB from Mikromasch) substrates and left overnight to allow the evaporation of water at a constant temperature (25 °C) and a relative humidity of 70 ± 5%. Samples produced in this way are coated with a thin water film, which can leave biomolecules in a hydrated state.

The AFM imaging of fibrillar α-Syn was performed in ambient conditions at room temperature (25 °C) using a JPK Nanowizard 3 Ultraspeed AFM from JPK instruments in amplitude modulation AFM (AM-AFM) with low force settings (80–90% of the free amplitude A ~20 nm). In AM-AFM modulation, the tip-surface distance regulation is performed to maintain constant the amplitude to a precised setpoint. Gold coated silicon cantilevers PPP-NCH-AuD (Nanosensors, Neuchâtel, Switzerland) with a spring constant of ~42 N/m and a tip curvature radius of ~10 nm have been used.

For each sample, a large number of fibrillar assemblies were systematically imaged at high resolution (5 μm × 5 μm, 1024 × 1024 pixels) with AFM and their morphology (height, width and periodicity) were analyzed manually with the JPK Data Processing software (JPK Instruments) using the line profile measurement option. For fibrillar assemblies showing periodicities, only the maximum fibril height was included in the height distribution analysis.

For fibrillar assembllies width determination and in order to reduce the tip convolution effect, ultrasharp AFM cantilevers Hi’Res-C15/Cr-Au-5 with a tip curvature of ~1 nm (Mikromasch) and a spring constant of ~42 N/m was used in phase modulation AFM (PM-AFM). In PM-AFM, the probe is oscillated in constant amplitude mode using an Automatic Gain Control (AGC) of the oscillation amplitude. The phase shift in the probe relative to the excitation signal is thus used as the feedback signal for the tip-sample distance z control, and the surface topography is obtained by measuring the z position required to keep the phase shift constant. The image size in this case was not higher than 1 μm × 1 μm (pixel size 0.95 nm) to preserve the tip sharpness and only few numbers of images at high resolution (1 μm × 1 μm, 1024 × 1024 pixels) have been performed with the same tip to minimize tip convolution effect. In PM-AFM, the constant amplitude was kept at ~14 nm and the phase setpoint was 1°.

### Persistence length and bending rigidity calculation

For the persistence length analysis (P_*l*_), we have imported the AFM heightmaps into open source software “Easyworm” written on Matlab which was developed by Lamour et *al.*^25^. This software allows the tracking of the fibrils contour length and then the calculation of their persistence length by analyzing the shape fluctuation of the fibrils. Only intact and isolated fibrils of at least 600 nm in length were included in the analysis. The calculation of the P_*l*_ can be done using three different expressions (decay of tangent-tangent correlations 〈cos θ〉, mean-squared end-to-end distance 〈R^2^〉_2D_ and mean square of the deviations 〈δ^2^〉_2D_ to secant midpoints as a function of the distance) all derived from Worm-like chain (WLC) model for semi-flexible polymers^25^. However, only the mean-squared end-to-end distance 〈R^2^〉_2D_ provided the best fits to the experimental fits with a coefficient of determination^25^ >0.9.

The mean-squared end-to-end distance 〈R^2^〉_2D_ for a worm-like chain (WLC) model is dependent on the internal contour length *l* in 2D: and it is expressed as follows^25^,^29^:


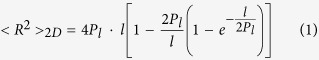


From the persistence length, the bending rigidity (κ) was calculated using the following equation:


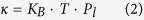


where T is the room temperature (298 °K) and K_B_ is the Boltzmann constant.

### Young’s modulus calculation

The Young’s modulus (E) is calculated from the bending rigidity using the following equation:


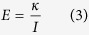


where *I* is the second moment of area (or moment of inertia). The latter was calculated using the ellipsoidal model of fibrils structure corresponding to the geometry of the cross-sectional area of an ellipsoidal shape. An ellipsoidal geometry was attributed to fibrils since their height (h) was lower than their width (w) albeit the use of ultrasharp probes. Two second moments of area, with respect to the two main axis of symmetry, result from our assumption: i) the first one is normal to the substrate and ii) the second is parallel to the substrate. However, as the bending energy is proportional to the thermal energy (K_B_.T) needed to bend the fibrils, it reflects the lowest energy bending mode that corresponds to the one obtained with the lowest moment of inertia. The lowest moment of area (*I*) of an ellipsoidal shape is thus the one in the direction of fibrils height (i.e. normal to the substrate) and it is given as follows^29^.


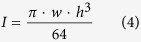


However, as the polymorph named ribbons exhibited a “tape-like” shape with a rectangular cross section, we calculated the second moment of area using the tape/ribbon like model^25^,^29^:


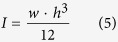


## Additional Information

**How to cite this article**: Makky, A. *et al*. Nanomechanical properties of distinct fibrillar polymorphs of the protein α-synuclein. *Sci. Rep.* **6**, 37970; doi: 10.1038/srep37970 (2016).

**Publisher's note:** Springer Nature remains neutral with regard to jurisdictional claims in published maps and institutional affiliations.

## Supplementary Material

Supplementary Information

## Figures and Tables

**Table 1:** Morphological and mechanical properties of the different fibrils type determined on either mica or HOPG substrates.

Sample	Fibrils	Ribbons	Fibrils-65	Fibrils-91
**Mica substrate**				
**Height (nm)**	6.4 ± 0.7	5.1 ± 0.8	6.7 ± 0.8	5.4 ± 1.5
	(n = 110)	(n = 113)	(n = 144)	(n = 210)
**Width (nm)**	15.0 ± 0.9	20.1 ± 0.7	21.9 ± 1.8	20.6 ± 1.7
	(n = 61)	(n = 53)	(n = 45)	(n = 52)
**Measured fibril length (μm)**	1.4 ± 0.5	1.1 ± 0.6	1.9 ± 0.7	1.7 ± 0.9
	(n = 58)	(n = 73)	(n = 112)	(n = 56)
**Persistence length (μm)**	14.2 ± 3.3	3.5 ± 1.0	6.5 ± 3.2	5.8 ± 1.7
	(n = 58)	(n = 73)	(n = 112)	(n = 56)
**Bending rigidity (×10**^**−26**^**N.m**^**2**^)	5.8 ± 1.3	1.4 ± 0.4	2.7 ± 1.3	2.4 ± 0.7
**Second moment of area (×10**^**−34**^**m**^**4**^**) of ellipsoidal shape**	1.9	1.3	3.2	1.6
**Young modulus (GPa) of ellipsoidal shape**	0.30	0.11	0.08	0.15
**Second moment of area (×10**^**−34**^**m**^**4**^**) of tape shape**	3.3	2.2	5.4	2.7
**Young modulus (GPa) of tape shape**	0.17	0.06	0.05	0.08
**HOPG substrate**				
**Persistence length (μm)**	12.6 ± 3.3	4.1 ± 1.3	6.5 ± 1.0	5.5 ± 2.0
	(n = 53)	(n = 61)	(n = 56)	(n = 38)
**Bending rigidity (×10**^**−26**^**N.m**^**2**^)	5.2 ± 1.3	1.7 ± 0.6	2.7 ± 0.4	2.3 ± 0.8
