# Supplementary material for: Nanomechanical properties of distinct fibrillar polymorphs of the protein α-synuclein
Source: Sci Rep. 2016 Nov 30;6:37970. doi: 10.1038/srep37970 (PMC5128817; doi:10.1038/srep37970)
Supplement: Supplementary Information [file srep37970-s1.doc]

**Supplementary Information**

**Nanomechanical properties of distinct fibrillar polymorphs of the protein α-synuclein**

Ali Makky ^1^*, Luc Bousset ^2^, Jérôme Polesel ^3^ and Ronald Melki ^2^ *

1. Institut Galien Paris-Sud, CNRS, Univ. Paris-Sud, University Paris-Saclay, 92296 Châtenay-Malabry, France.

2. Paris-Saclay Institute of Neuroscience, Centre National de la Recherche Scientifique, Université Paris-Saclay, 91190 Gif-sur-Yvette, France.

3. **Luxembourg Institute of Science and Technology (LIST), Materials Research and Technology (MRT), L-4422 Belvaux, Luxembourg.**

* Corresponding authors: ali.makky@u-psud.fr, luc.bousset@cnrs.fr

**Figure S1:** Width of fibrils, ribbons, fibrils-65 and fibrils-91 derived from negatively stained TEM acquired at a magnification 10000x (pixel size of 0.66nm) 80 cross-sections were analyzed with Image J (Plot profile option). TEM magnification calibration was performed with calibration grating at 10000x.

**Figure S2:** PM-AFM topography images (a) and height profile (b) of α-syn fibrils obtained with ultrasharp probe. Compared to the noise level measured on mica, the line profile of α-syn fibril suggests irregular morphological features along the fibril height.


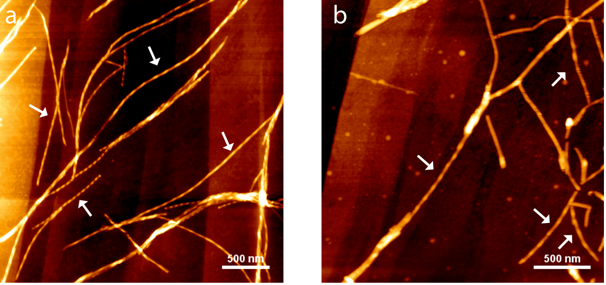


**Figure S3:** AM-AFM topography images in air of α-syn fibrils-65 (a) and -91 (b) adsorbed onto HOPG substrate. As upon adsorption to mica substrate, α-syn fibrils-65 show different pitch periodicity (white arrows) whereas α-syn fibrils-91 show an homogenous one.


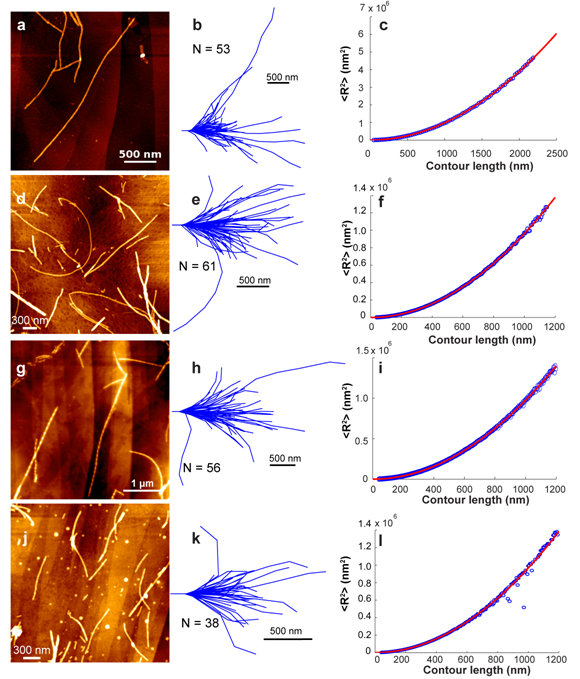


**Figure S4:** AM-AFM topography images in air of α-syn fibrils, ribbons, fibrils-65 and fibrils-P91, respectively adsorbed onto HOPG substrate (a,d,g,j). Contours of α-syn fibrils, ribbons, fibrils-65 and fibrils-P91, respectively (b,e,h,k). End-to-end distance (R^2^) plots as a function of contour length for four types of fibrils, ribbons, fibrils-65 and fibrils-P91 (c,f,i,l), respectively (blue open circles). Least-square fits are shown as red lines.
